# Supplementary material for: Inflating bacterial cells by increased protein synthesis
Source: Mol Syst Biol. 2015 Oct 30;11(10):836. doi: 10.15252/msb.20156178 (PMC4631207; doi:10.15252/msb.20156178)
Supplement: Supplementary file 1 — Appendix [file msb0011-0836-sd1.pdf]

Appendix for

Inflating bacterial cells by increased protein synthesis

Markus Basan *et al.*

This file includes:

Appendix Tables S1-S4
Appendix Figs. S1-S10
Appendix References

Appendix Tables

Growth limiting conditions	Strain	Growth conditions	Symbol
Nutrient limitation	NCM3722	RDM+glucose	● (green)
		Glucose+cAA	
		Glucose	
		Glycerol	
		Acetate	
		Mannose	
Translation inhibition with Cm	NCM3722	Glucose+2 μM Cm	▲ (blue)
		Glucose+4 μM Cm	
		Glucose+6 μM Cm	
		Glucose+8 μM Cm	
Glucose LacZ OE	NQ1389	Glucose+0 ng/mL cTc	◆ (red)
		Glucose+2.5 ng/mL cTc	
		Glucose+5 ng/mL cTc	
		Glucose+10 ng/mL cTc	
		Glucose+15 ng/mL cTc	
Glucose+cAA LacZ OE	NQ1389	Glucose+cAA+0 ng/mL cTc	◇ (red)
		Glucose+cAA+2.5 ng/mL cTc	
		Glucose+cAA+5 ng/mL cTc	
		Glucose+cAA+10 ng/mL cTc	
		Glucose+cAA+15 ng/mL cTc	
		Glucose+cAA+20 ng/mL cTc	

Appendix Table S1-Summary of growth conditions in this study. Every set of data presented in this work was collected in a separate experiment using the same strains and identical growth conditions.

Abbreviations : RDM+glucose – MOPS buffered rich defined medium+0.2% (w/v) glucose;

Glucose+cAA – MOPS+ 0.2 % casamino acids+0.2% (w/v) glucose; Glucose – MOPS+0.2%(w/v) glucose; Glycerol – MOPS + 0.2% (v/v) glycerol; Acetate – MOPS + 60 mM sodium acetate; Mannose – MOPS + 0.2% (w/v) mannose. Cm – chloramphenicol ; cTc – chlortetracycline; OE – overexpression

Strain	Medium	Growth rate (1/h)	cell length (μm)		cell width (μm)		cell volume (μm^3)	
			μ	σ	μ	σ	μ	σ
NCM3722	RDM+glucose	1.83	4.94	0.84	1.36	0.11	6.6	2.3
	Glucose+cAA	1.29	3.29	0.49	1.21	0.07	3.33	0.61
	Glucose	0.98	2.95	0.45	1.07	0.06	2.32	0.45
	Glycerol	0.70	2.84	0.51	0.92	0.07	1.7	0.43
	Acetate	0.47	2.51	0.42	0.98	0.08	1.67	0.61
	Mannose	0.42	2.37	0.41	0.91	0.06	1.36	0.34
	Glucose+2 μM Cm	0.74	2.72	0.41	1.11	0.05	2.28	0.45
	Glucose+4 μM Cm	0.55	2.74	0.46	1.09	0.05	2.23	0.47
	Glucose+6 μM Cm	0.45	2.88	0.47	1.11	0.05	2.42	0.50
	Glucose+8 μM Cm	0.30	3.00	0.52	1.08	0.06	2.44	0.58
NQ1389	Glucose+ 0 ng/mL cTc	0.88	3.15	0.58	1.1	0.05	2.67	0.62
	Glucose+ 2.5 ng/mL cTc	0.73	3.15	0.50	1.13	0.05	2.79	0.57
	Glucose+ 5 ng/mL cTc	0.61	3.47	0.59	1.15	0.08	3.22	1.00
	Glucose+ 10 ng/mL cTc	0.40	4.89	1.08	1.23	0.08	5.39	1.72
	Glucose+ 15 ng/mL cTc	0.24	5.92	1.08	1.27	0.11	7.13	2.46
	Glucose+cAA +0 ng/mL cTc	1.12	3.11	0.39	1.21	0.07	3.11	0.58
	Glucose+cAA +2.5 ng/mL cTc	0.95	3.13	0.47	1.22	0.07	3.23	0.72
	Glucose+cAA +5 ng/mL cTc	0.84	3.27	0.53	1.24	0.07	3.52	0.76
	Glucose+cAA +10 ng/mL cTc	0.65	3.51	0.69	1.32	0.07	4.24	1.16
	Glucose+cAA +15 ng/mL cTc	0.52	4.66	0.81	1.34	0.09	6.05	1.81
	Glucose+cAA +20 ng/mL cTc	0.42	5.76	0.92	1.36	0.08	7.61	2.21

Appendix Table S2-Cell size of NCM3722 and NQ1389 in various growth conditions. For every growth condition, cell length (L), cell width (W) and cell volume (V) of 500 to 1000 individual cells were measured. Cell length (L) and cell width (W) were directly extracted with the processing of ImageJ software. Cell volume (V) was calculated according to the equation, $V = \pi R^2 \cdot (L - 2R/3)$. The table gives the mean (μ) as well as the standard deviation (σ) of cell length, cell width and cell volume of the measured distribution cell sizes. Cell size distributions are presented in Fig. 1B for several exemplary conditions.

Strain	Medium	Growth rate (1/h)	cell length (µm)		cell width (µm)		cell volume (µm^3)	
		µ	µ	σ	µ	σ	µ	σ
NCM3722	Acetate (1st)	0.47	2.51	0.42	0.98	0.08	1.67	0.61
	Acetate (2nd)	0.46	2.54	0.41	0.98	0.06	1.67	0.34
	**Acetate Mean (STD)**	**0.46 (0.00)**	**2.53 (0.01)**	**0.42 (0.00)**	**0.98 (0.00)**	**0.07 (0.01)**	**1.67 (0.00)**	**0.48 (0.14)**
	Glucose+CAA(1st)	1.28	3.15	0.49	1.24	0.07	3.30	0.72
	Glucose+CAA(2nd)	1.29	3.29	0.49	1.21	0.07	3.33	0.61
	**Mean (STD)**	**1.28 (0.01)**	**3.22 (0.07)**	**0.49 (0.00)**	**1.22 (0.01)**	**0.07 (0.00)**	**3.32 (0.02)**	**0.67 (0.05)**
NQ1389	Glucose+10 ng (1st)	0.40	4.89	1.08	1.23	0.08	5.39	1.72
	Glucose+10 ng (2nd OD 0.35)	0.41	4.94	1.08	1.21	0.06	5.23	1.84
	Glucose+10 ng (2nd OD 0.46)	0.41	4.84	1.21	1.20	0.08	5.17	1.88
	Glucose+10 ng (2nd OD 0.54)	0.41	4.84	1.20	1.24	0.07	5.43	1.83
	Glucose+10 ng (3rd OD 0.23)	0.39	4.72	1.17	1.24	0.07	5.32	1.77
	Glucose+10 ng (3rd OD 0.46)	0.39	4.81	1.18	1.26	0.07	5.52	1.85
	**Glucose+10 ng Mean (STD)**	**0.40 (0.01)**	**4.84 (0.07)**	**1.15 (0.05)**	**1.23 (0.02)**	**0.07 (0.01)**	**5.34 (0.12)**	**1.81 (0.06)**
	Glucose+15 ng(1st)	0.24	5.92	1.08	1.27	0.11	7.13	2.46
	Glucose+15 ng (2nd)	0.23	5.71	1.16	1.28	0.09	6.89	2.30
	Glucose+15 ng (3rd OD 0.33)	0.23	5.96	1.38	1.25	0.09	6.92	2.28
	Glucose+15 ng (3rd OD 0.46)	0.23	6.15	1.73	1.29	0.09	7.63	3.11
	Glucose+15 ng (4th OD 0.23)	0.24	5.78	1.46	1.31	0.09	7.25	2.69
	Glucose+15 ng (4th OD 0.45)	0.24	5.87	1.43	1.32	0.09	7.49	2.61
	**Glucose+15 ng Mean (STD)**	**0.24 (0.00)**	**5.90 (0.14)**	**1.37 (0.21)**	**1.29 (0.02)**	**0.09 (0.01)**	**7.22 (0.27)**	**2.57 (0.28)**

Appendix Table S3-Reproducibility of cell size measurements in biological repeats and various cell culture densities (OD_{600}). The table gives the mean (µ) as well as the standard

deviation (σ) of cell length, cell width and cell volume of the measured distribution cell sizes for multiple biological repeats (1st, 2nd, etc.) repeats and at various culture densities (OD_{600}). Mean and standard deviations (STD) of the distribution properties of the combined biological repeats and measurements at different optical density are also presented.

Strain	Medium	Growth rate (1/h)	DNA(μg)/OD_{600}	RNA(μg)/OD_{600}	Protein(μg)/OD_{600}	Dry mass (μg)/OD_{600}	Cell Number (10^8)/OD_{600}
NCM3722	RDM+glucose	1.84 ± 0.02	11.1 ± 0.66	153 ± 8.43	329 ± 13.9	507	3.43 ± 0.16
	Glucose+cAA	1.27 ± 0.02	11.3 ± 0.60	115 ± 2.2	317 ± 16.3	500	7.93 ± 0.73
	Glucose	0.98 ± 0.01	11.9 ± 0.67	97.9 ± 2.37	333 ± 11.5	494	10.7 ± 0.69
	Glycerol[a]	0.7 ± 0.01	14.1 ± 0.87	83.4 ± 3.3	355 ± 16.8	522	16.0 ± 0.98
	Acetate[a]	0.45 ± 0.01	14.2 ± 0.68	64.5 ± 5.9	378 ± 3.39	516	17.1 ± 1.48
	Mannose[a]	0.42 ± 0.01	16.5 ± 0.49	66.1 ± 1.4	391 ± 8.32	513	19.4 ±0.84
	Glucose + 2 μM Cm	0.75 ± 0.01	13.9 ± 0.38	114 ± 2.66	339 ± 10.9	510	12.6 ± 0.27
	Glucose + 4 μM Cm	0.55 ± 0.02	15.4 ± 0.58	127 ± 3.29	309 ± 5.89	507	13.3 ± 0.50
	Glucose + 6 μM Cm	0.42 ± 0.00	15.9 ± 0.34	135 ± 6.15	300 ± 12.2	524	12.5 ± 0.59
	Glucose + 8 μM Cm	0.32 ± 0.01	16.6 ± 0.42	143 ± 1.22	288 ± 6.68	494	12.3 ± 0.69
NQ1389	Glucose + 0 ng/mL cTc	0.88 ± 0.00	12.1 ± 0.55	91.3 ± 2.92	340 ± 19.4	475	9.60 ± 0.17
	Glucose + 2.5 ng/mL cTc	0.72 ± 0.02	11.1 ± 0.72	77.1 ± 1.81	307 ± 8.08	458	8.07 ± 0.11
	Glucose + 5 ng/mL cTc	0.58 ± 0.02	9.47 ± 0.60	65.8 ± 0.36	326 ± 13	440	6.14 ± 0.07
	Glucose + 10 ng/mL cTc	0.35 ± 0.02	7.69 ± 0.66	46.6 ± 1.62	304 ± 14.2	414	3.16 ± 0.01
	Glucose + 15 ng/mL cTc	0.24 ± 0.00	6.67 ± 0.28	38.6 ± 0.16	321 ± 11.3	393	2.14 ± 0.01
	Glucose+cAA + 0 ng/mL cTc	1.1 ± 0.03	11.3 ± 0.91	101 ± 3.17	313 ± 24.7	482	6.99 ± 0.17
	Glucose+cAA +2.5 ng/mL cTc	0.95 ± 0.01	11.1 ± 0.81	95.4 ± 4.84	334 ± 15.9	472	5.63 ± 0.11
	Glucose+cAA + 5 ng/mL cTc	0.85 ± 0.01	10.7 ± 0.43	93.3 ± 1.05	322 ± 19.4	466	5.38 ± 0.12
	Glucose+cAA + 10 ng/mL cTc	0.64 ± 0.01	10.1 ± 0.48	78.6 ± 6.9	344 ± 17	458	3.58 ± 0.05
	Glucose+cAA + 15 ng/mL cTc	0.51 ± 0.01	9.28 ± 0.31	73.6 ± 3.43	328 ± 10.8	448	2.71 ± 0.08
	Glucose+cAA + 20 ng/mL cTc	0.42 ± 0.01	8.85 ± 0.54	65.1 ± 1.58	335 ± 8.57	446	2.33 ± 0.02

Appendix Table S4-Growth rate, macromolecular content, and cell count of WT and LacZ OE strains in various growth conditions. Raw data were generated according to Materials and Method and plotted in Fig. EV2, Appendix Figure S8. Cell no/OD_{600} for NCM3722 growing in the three conditions (glycerol, acetate, mannose) were determined by plating, because the small cell sizes in these three conditions were too close to the detection limit of the Coulter counter; see Fig. EV2.

Appendix Figures

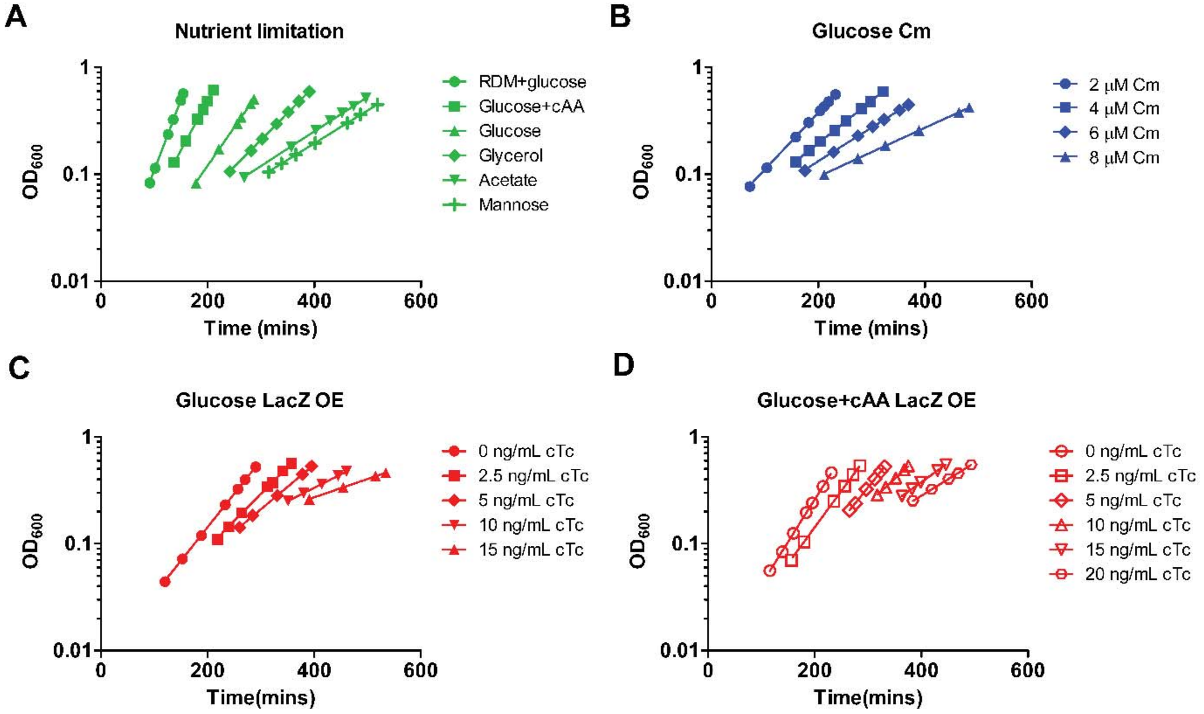

Appendix Figure S1: Growth curves for the different growth limitations. Corresponding growth curves for the cultures analyzed in this work. For LacZ OE, the inducer cTc was added during initial exponential growth (at OD_{600} around 0.05) to prevent the occurrence of mutation. Growth curves are plotted 2-3 doublings after inducer addition after cultures have reached their new exponential growth rates.

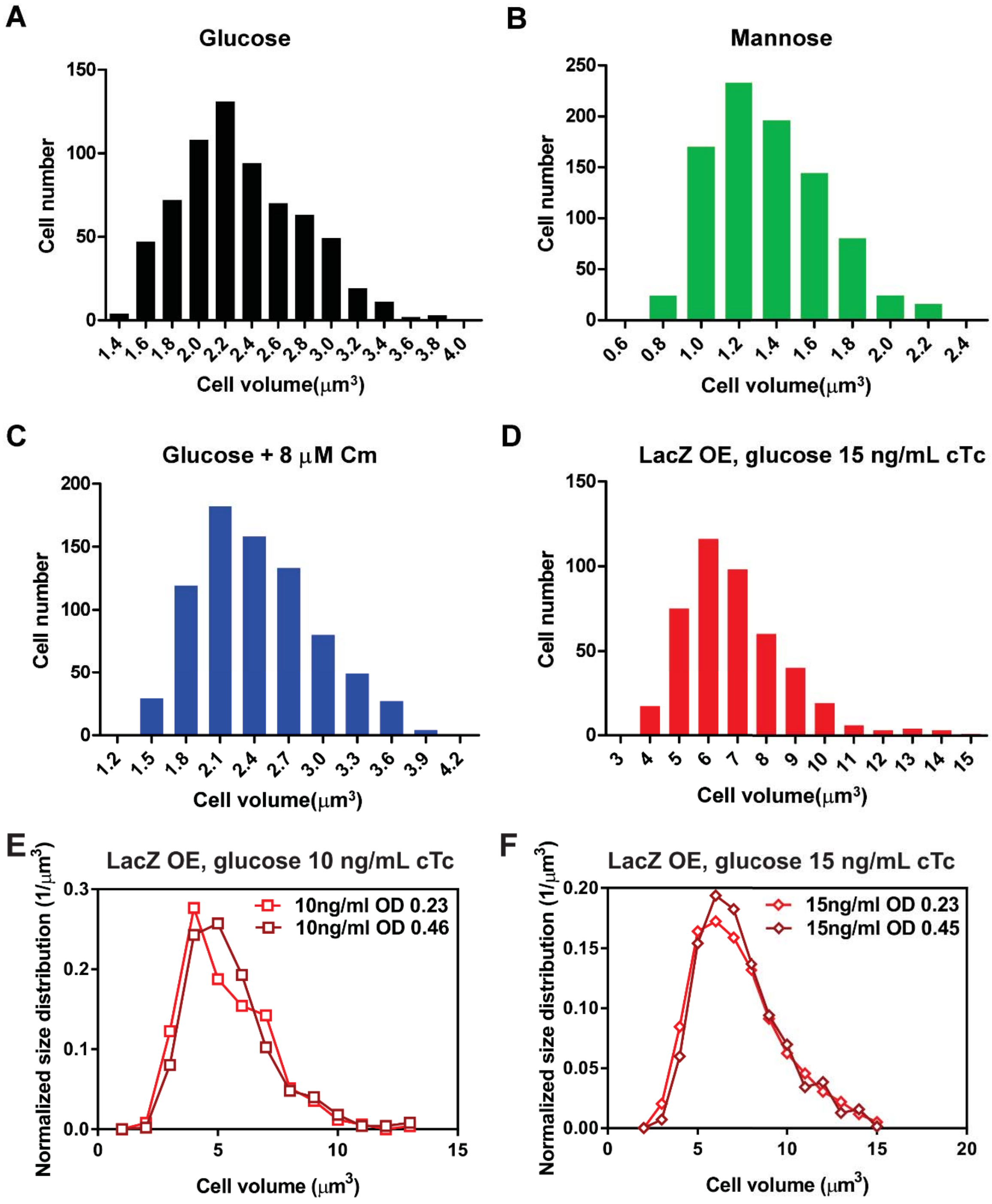

Appendix Figure S2: Cell size distribution in exemplary conditions for the different growth limitations. Cell size distributions in the exemplary conditions presented in Fig. 1A of the main text. **A,** Glucose ($\lambda \approx 0.98$/hr); **B,** Mannose ($\lambda \approx 0.41$/hr); **C,** Glucose + 8 μM Cm ($\lambda \approx 0.32$/hr); **D,** LacZ OE, glucose + 15 ng/mL cTc ($\lambda \approx 0.25$/hr). See Appendix Table S2 for means and standard deviations of cell size distributions for all conditions studied. **E,** Normalized size distribution for LacZ OE, glucose + 10 ng/mL cTc at different OD_{600}. Size distributions measured at different

OD_{600} are very similar. **F,** Normalized size distribution for LacZ OE, glucose + 15 ng/mL cTc at different OD_{600} from a biological repeat of the condition presented in panel D (see Appendix Table S3). Size distributions measured at different OD_{600} are very similar.

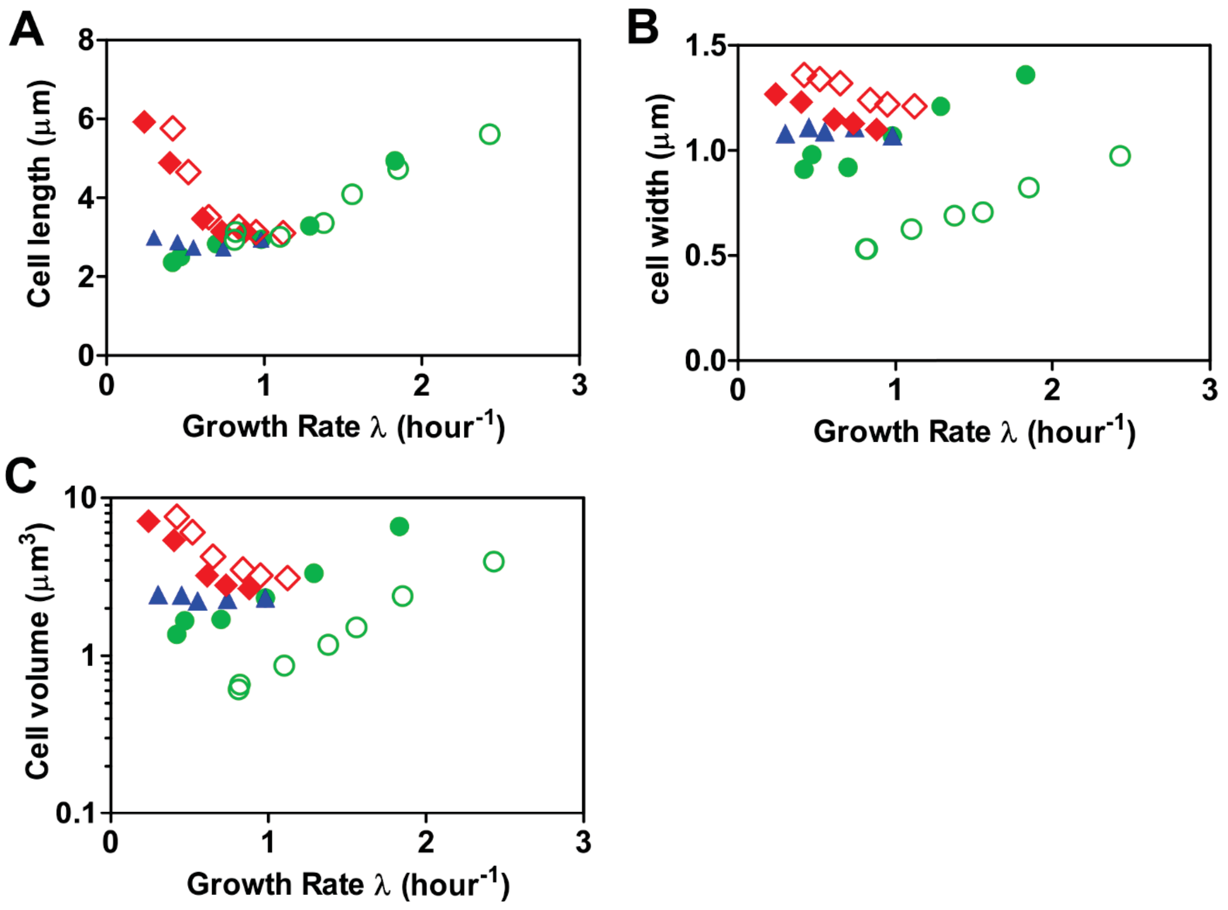

Appendix Figure S3: Cell length, width and volume for the different growth limitations. Averages and standard deviations of **A,** Cell length (*L*), **B**, cell width (*D*) and **C**, cell volume (*V*) measured by microscopy in our study (see Appendix Table S2 for means and standard deviations). Symbols used are as specified the same as in Appendix Table S1, except for the open green circles, which indicate data for nutrient-limited growth obtained from a recent single-cell study on the same strain by Taheri-Araghi *et al.* (Taheri-Araghi *et al*, 2015). Cell length in our study is very similar to the results of Taheri-Araghi *et al.* For cell width, our data exhibits a parallel shift of 0.4 µm to larger values as compared to Taheri-Araghi *et al.* This is caused by the uncertainty of cell width determination using optical microscopy, due to the difficulty in accurately defining the cell edge (0.2 µm difference on each side). As a result of the difference in cell width, cell volume obtained in our study, defined as $V = \pi R^2 \cdot (L - 2R/3)$ where R is half of the width, also shows a higher value compared to Taheri-Araghi *et al.* in nutrient limitated growth. Apart from this systematic deviation due to subtleties of cell width measurement, our results are in good agreement with the data in the study by Taheri-Araghi *et al.* (Taheri-Araghi *et al*, 2015).

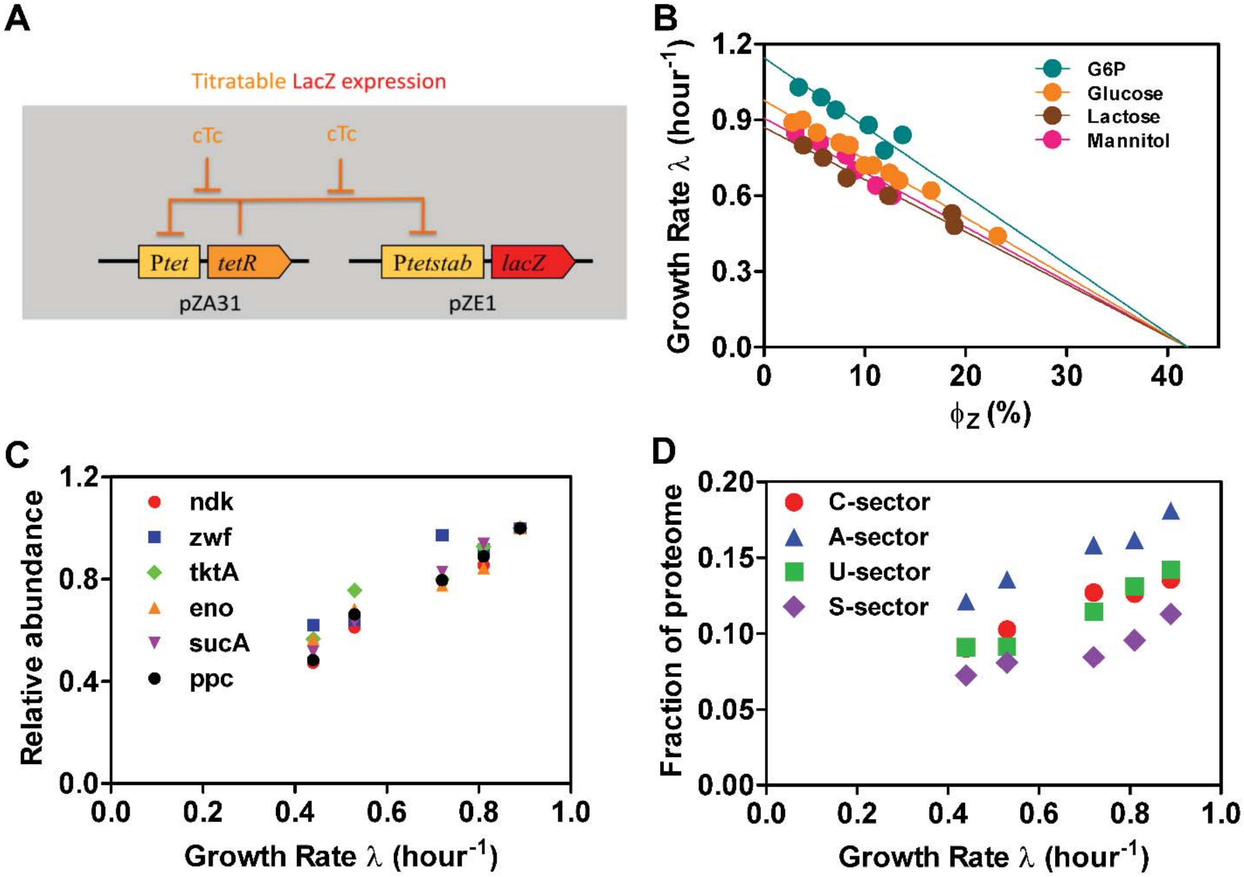

Appendix Figure S4: LacZ overexpression system. A, Schematic illustration of the construct used for LacZ OE. The strain carries two plasmids, pZE1 and pZA31. On the plasmid pZE1, LacZ is expressed from a stabilized version of the synthetic $P_{Ltet\text{-}O1}$-promoter described previously (Hui *et al*, 2015), while on the plasmid pZA31, the Tet-repressor, TetR, is expressed under the $P_{Ltet\text{-}O1}$-promoter. The addition of the inducer chloro-tetracycline (cTc) releases TetR from repressing the tet-promoter and increases the expression level of LacZ. This autorepression circuit allows a gradual, linear increase in the expression level of LacZ with increasing inducer concentration. Illustration taken from Basan *et al.* (Basan *et al*). **B,** Growth rate plotted against the level of LacZ OE (as a fraction of the total proteome) for different glycolytic carbon sources (different colors), presented in Basan *et al.* (Basan *et al*). For each carbon source, LacZ OE leads to a linear decrease in growth rate, as characterized by Scott *et al.* (Scott *et al*, 2010). **C,** Relative change of protein abundances for examplary proteins under LacZ OE as characterized in (Hui *et al*, 2015). The increasing proteome fraction occupied by LacZ compresses the proteome fraction of many groups of proteins, resulting in a direct proportionality of these protein levels with growth rate (Hui *et al*, 2015). The examples shown are selected from catabolic and anabolic proteins, and in particular, enzymes involved in DNA nucleotide synthesis (NDK). **D,** The response of different proteome sectors to LacZ OE, as defined and characterized by Hui *et al* (Hui *et al*, 2015). The different proteome sectors are approximately compressed uniformly by LacZ OE. The U-sector (green

squares) contain most of the enzymes of nucleotide synthesis pathways. The non-vanishing y-intercepts result from the fact that the abundances of some proteins in the sectors are not compressed to zero, i.e., with a non-zero y-offset, as the proteins presented in panel C.

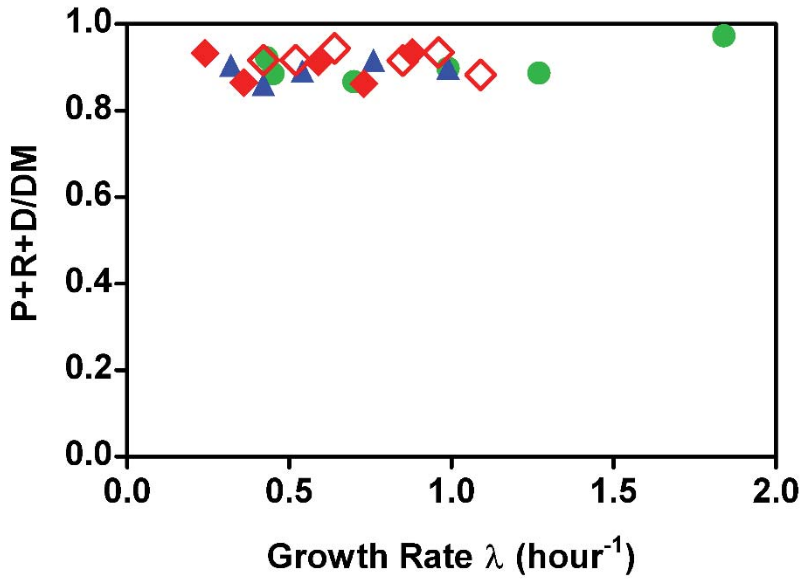

Appendix Figure S5: DNA+RNA+protein per dry mass. Dry mass composition under the three perturbations Symbols same as Appendix Table S1. The combined mass of DNA, RNA and protein per dry mass plotted against growth rate. Together, DNA, RNA and protein account for roughly 90% of total dry mass in all conditions.

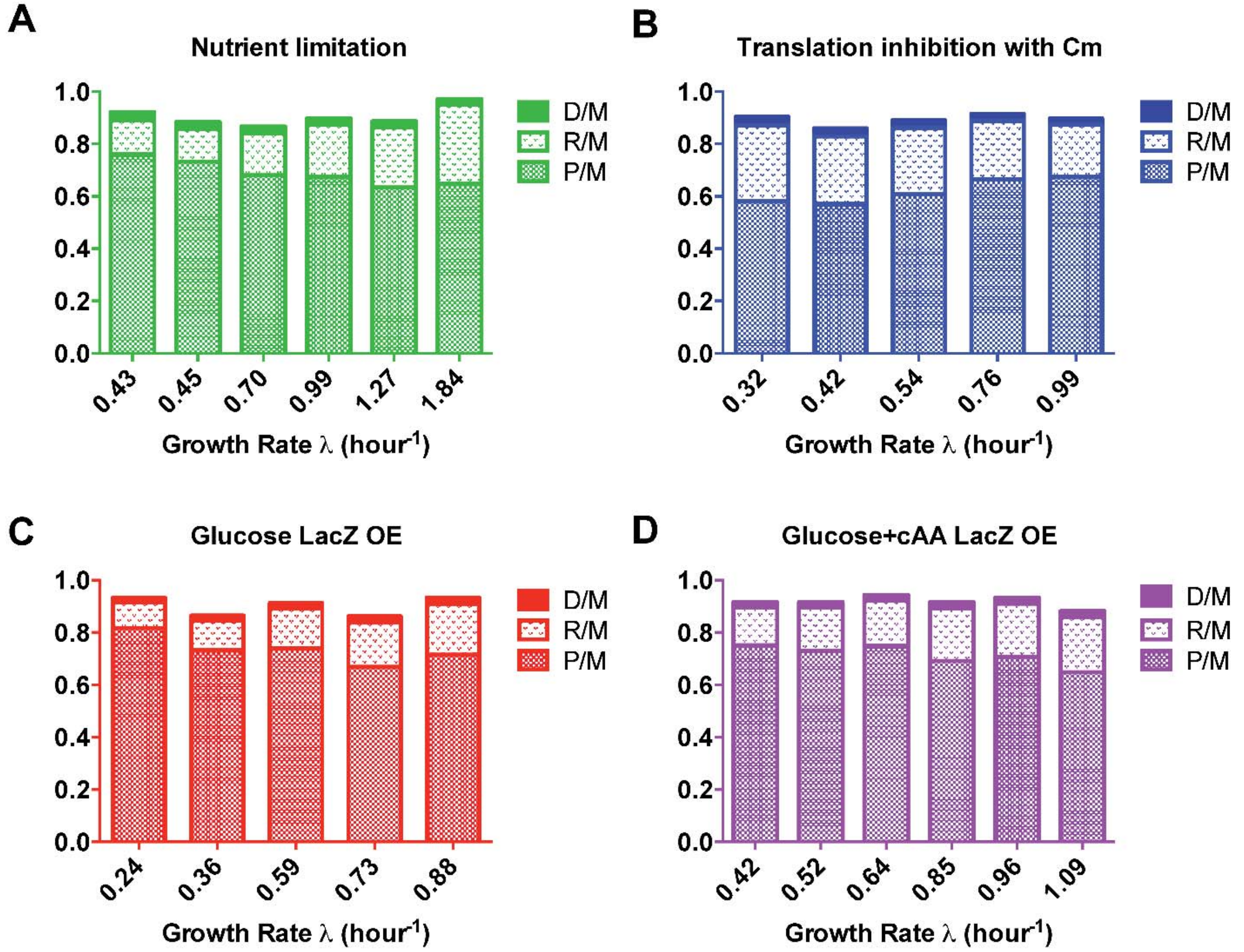

Appendix Figure S6: Dry mass composition under the three perturbations. Proteins, RNA and DNA as a fraction of total dry mass for the different limitations. **A,** Nutrient limitation. The increase the RNA fraction at fast growth rates is due to a higher ribosomal content required for fast growth (Scott *et al*, 2010). **B,** Chloramphenicol inhibition. Chloramphenicol leads to a lower ribosome efficiency and a corresponding increase of the ribosomal protein fraction at slow growth rates (Scott *et al*, 2010; Hui *et al*, 2015). This results in a higher RNA fraction of dry mass at slow growth rates. **C,** LacZ OE in glucose minimal medium. LacZ OE results in a larger protein fraction of dry mass at slow growth rates. **D,** LacZ OE in glucose+cAA minimal medium. Results are similar to panel C.

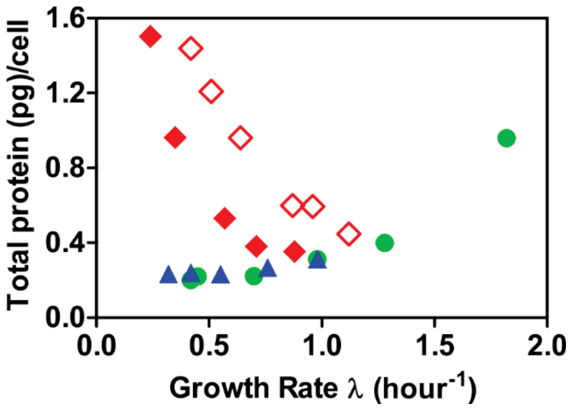

Appendix Figure S7: Cellular protein content for the different growth limitations. Symbols same as Appendix Table S1. Protein per cell determined from Appendix Table S4. Protein per cell dominates cellular dry mass and displays similar trends as cell size (see Fig. 1C&D).

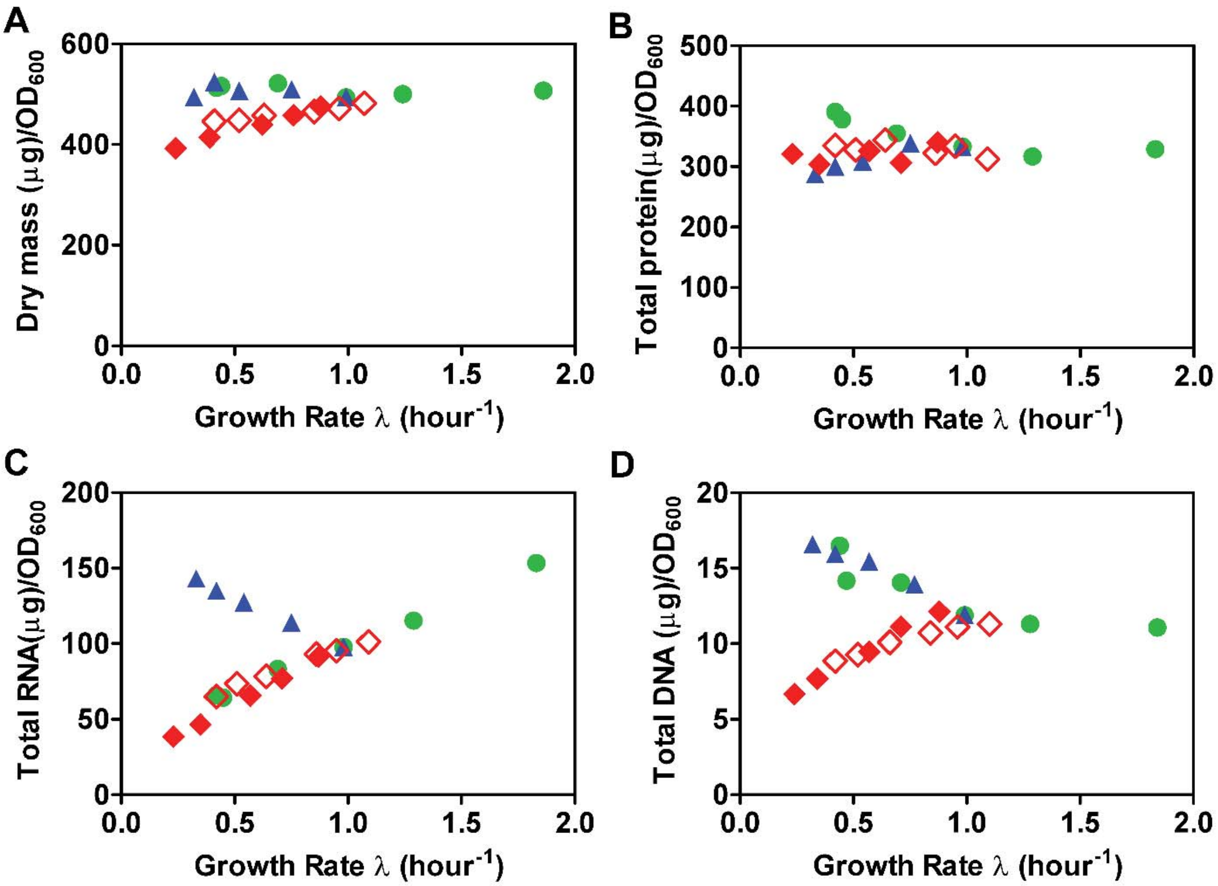

Appendix Figure S8: Raw data for the macromolecular composition of the culture. A, dry mass, **B,** total protein, **C,** total RNA and **D,** total DNA quantified per (ml*OD_{600}) of culture under the three modes of growth perturbations (data presented in Appendix Table S4). Symbols same as Appendix Table S1. In combination with the cell counts (Fig. EV2), this data was used to calculate per cell quantities throughout this work.

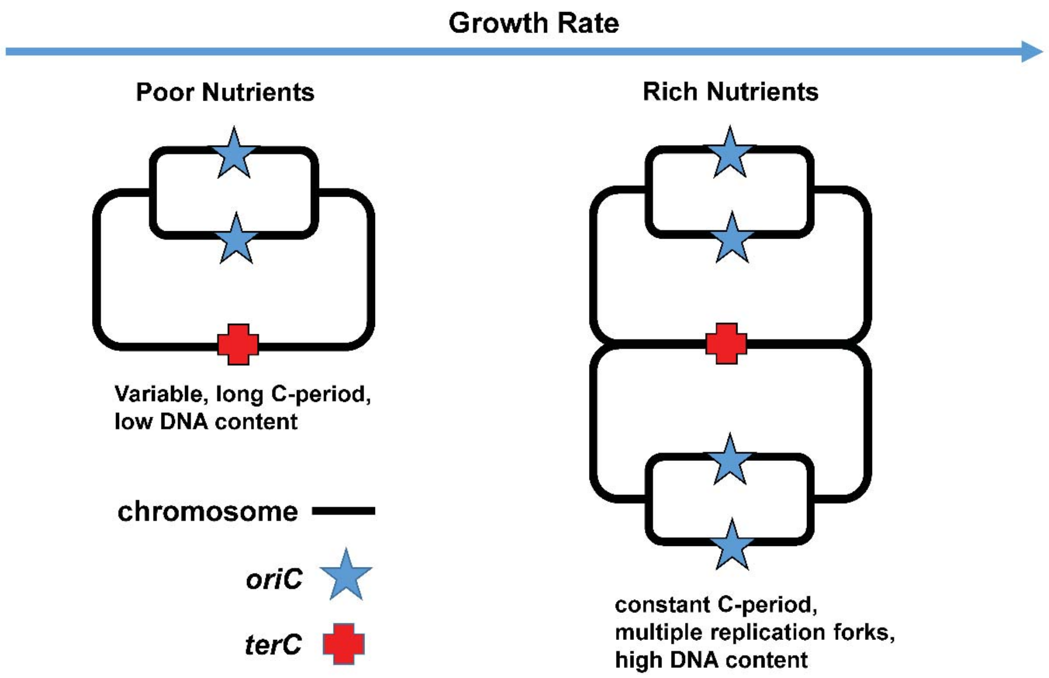

Appendix Figure S9: The Helmstetter-Cooper model of DNA replication for bacteria. Illustration adapted from Wang *et al.* (Wang & Levin, 2009). For slowly growing cells on a poor nutrient source (with DT > 60 min for WT cells), there is one replication fork present per cell. Each cell has one copy of the terminus (*terC*) and only two copies of the replication origin (*oriC*) when the chromosome is replicated. The duration of time needed to complete one round of chromosome replication, the C-period, increases with decreasing growth rate for nutrient-limited growth due to limitation in nucleotide (DNA monomer) synthesis (Neidhart, 1996). On the other hand, at fast growth rates (DT < 60 min), the C-period is constant (40 min) and the speed of replication is limited by the speed of DNA polymerase. Cell division occurs 20 min (D-period) after the completion of DNA replication. To enable growth with doubling times shorter than the C+D-period (60 min), multiple rounds of DNA replication occur simultaneously in a single cell. A new round of replication is initiated from each chromosome before the first round has terminated. This results in a chromosomal complex with multiple replication forks. Each cell still has only one copy of *terC*, but can have up to eight copies of the chromosomal region around *oriC*. LacZ OE constitutes a new regime within the Helmstetter-Cooper model of DNA replication. Despite very slow growth, cells exhibit high cellular DNA content in a single nucleoid complex (see Fig. EV3C). This implicates multiple replication forks in combination with a very long, variable C-period.

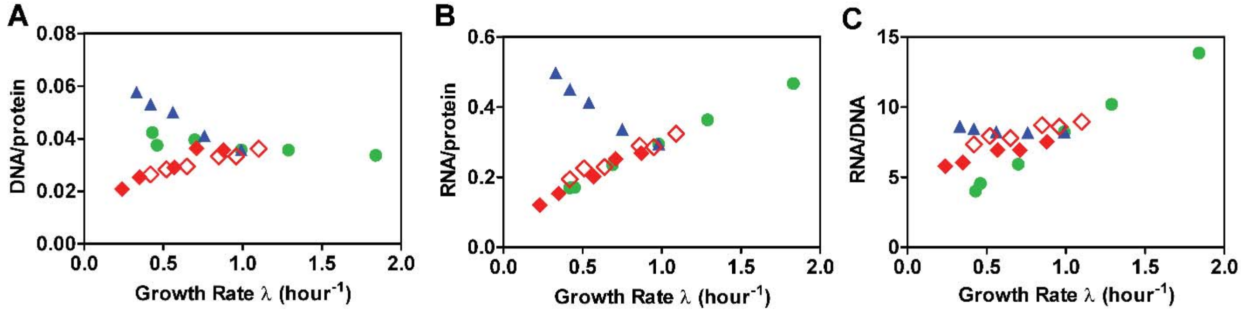

Appendix Figure S10: Ratio of the macromolecular compositions under different modes of growth perturbations. Symbols same as Appendix Table S1. **A,** DNA content per protein (D/P). Unlike commonly assumed, the DNA protein ratio $< D >/< P >$ is not constant, but changes by more than a factor 2 between the different perturbations with a decrease for protein overexpression and an increase under Cm inhibition. **B,** RNA per protein ratio $< R >/< P >$. $< R >/< P >$ is a proxy for ribosomal content, as most cellular RNA is ribosomal RNA (Scott *et al*, 2010). The growth rate dependence of this ratio follows from microbial growth laws (Scott *et al*, 2010), where ribosomal content is proportional to growth rate for carbon limitation as well as LacZ OE and increases under chloramphenicol inhibition, because the efficiency of ribosomes is decreased. **C,** RNA/DNA ratio $< R >/< D >$. $< R >/< D >$ is a proxy for the ratio of ribosomes to DNA. $< R >/< D >$ exhibits an increase with growth rate under carbon limitation, while for LacZ OE and Cm inhibition $< R >/< D >$ is roughly constant. The change in $< P >/< D >$ reported in A, results from the combination of $< R >/< P >$ reported in B, given by growth laws and the change in $< R >/< D >$ reflecting the transcriptional activity of ribosomal promoters.

Appendix References

Basan M, Hui S, Zhang Z, Shen Y, Williamson JR & Hwa T Overflow metabolism in bacteria results from efficient proteome allocation for energy biogenesis. *Nature*

Hui S, Silverman JM, Chen SS, Erickson DW, Basan M, Hwa T & Williamson JR (2015) Quantitative proteomic analysis reveals a simple strategy of global resource allocation in bacteria. *Mol. Syst. Biol.*

Neidhart FC (1996) Escherichia coli and Salmonella.

Scott M, Gunderson CW, Mateescu EM, Zhang Z & Hwa T (2010) Interdependence of cell growth and gene expression: origins and consequences. *Science* **330:** 1099–102

Taheri-Araghi S, Bradde S, Sauls JT, Hill NS, Levin PA, Paulsson J, Vergassola M & Jun S (2015) Cell-size control and homeostasis in bacteria. *Curr. Biol.* **25:** 385–91

Wang JD & Levin PA (2009) Metabolism, cell growth and the bacterial cell cycle. *Nat. Rev. Microbiol.* **7:** 822–7
