# Supplementary material for: Inflating bacterial cells by increased protein synthesis
Source: Mol Syst Biol. 2015 Oct 30;11(10):836. doi: 10.15252/msb.20156178 (PMC4631207; doi:10.15252/msb.20156178)
Supplement: Supplementary file 2 — Expanded View Figures PDF [file msb0011-0836-sd2.pdf]

Expanded View Figures

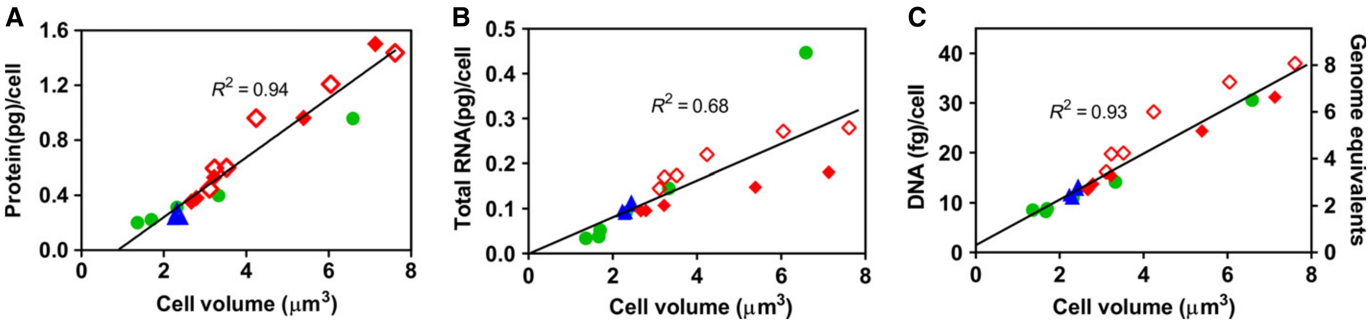

Figure EV1. Protein/RNA/DNA-volume correlation.

Symbols same as in Fig 1.

A, B Total cellular protein (A) and total cellular RNA (B) determined from Appendix Table S4, plotted against cell volume determined via microscopy, for each growth perturbation. The resulting direct proportionalities indicate that cell size closely corresponds to cellular protein under all growth limitations. This is related to the close correspondence between cell size and cellular dry weight (Fig 2E), because cellular protein is the main constituent of cellular dry mass (Appendix Fig S6).

C Cellular DNA plotted against cell volume determined via microscopy, for each growth perturbation. Remarkably, cellular DNA content closely follows the trends exhibited by cell size under all perturbations.

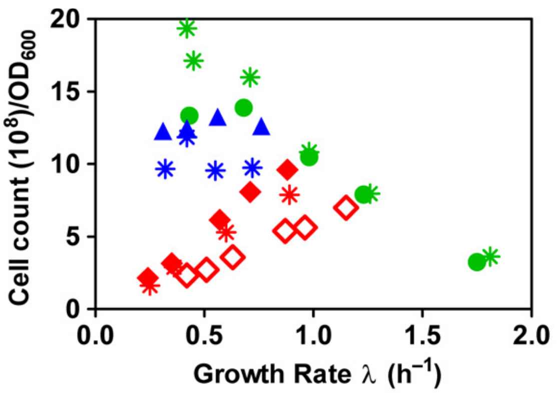

Condition	Method of cell count	Symbol
Nutrient limitation	Coulter counter	●
Nutrient limitation	Plating	*
Translation inhibtion with Cm	Coulter counter	▲
Translation inhibtion with Cm	Plating	*
Glucose LacZ OE	Coulter counter	◆
Glucose LacZ OE	Plating	*
Glucose+cAA LacZ OE	Coulter counter	◇

Figure EV2. Cell counts from Coulter counter and plating.

Cell counts for the three types of growth limitations were obtained by using a Coulter counter on the one hand and plating on the other hand. In general, there is a very good agreement between the results of the Coulter counter and plating counts. For LacZ overexpression (OE) in glucose minimal medium, plating (red asterisks) resulted in only slightly lower values (10%) than the Coulter counter method (filled red diamonds), demonstrating that almost all cells in the culture are viable even at large cell sizes. For the nutrient limitation series, the results of the two methods give good agreement except for the very slow growth conditions (GR < 0.7 h^{-1}), where the Coulter counter method (green filled circles) resulted in a lower cell number as compared to plating (green asterisks). This results from the small cell sizes under these conditions, which are close to the detection limit of the Coulter counter. For translation inhibition with Cm, the values of plating (blue asterisks) are lower compared to those obtained from the Coulter counter method (filled blue triangles) by 20%. This indicates that a fraction of cells in these conditions is not viable when subjected to plating. Throughout this manuscript, we use the cell count in order to determine per cell quantities. In these calculations, due to the small deviations discussed above, for the actual cell count, we employ the values of plating for nutrient limitation at slow growth rates, but use the values of the Coulter counter methods for both translation inhibition and LacZ overexpression.

 © 2015 The Authors

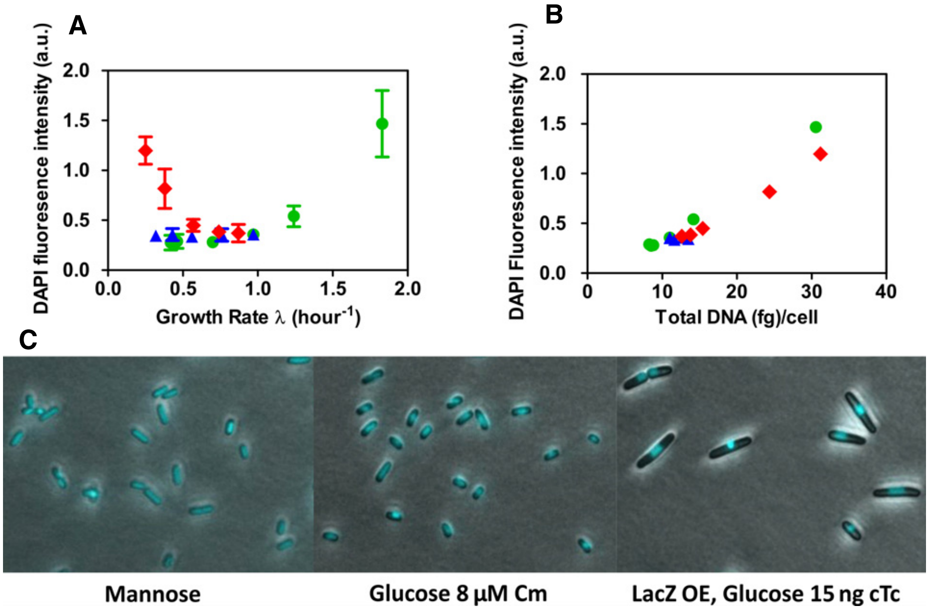

Figure EV3. Cellular DNA content from DAPI staining.

Symbols same as in Fig 1.

A Cellular DNA content measured by DAPI staining and microscopy plotted against growth rate. The results confirm the data for cellular DNA content (Fig 2F). Error bars represent the standard deviations of the measured distributions over the cell population ($n = 20$).

B Cellular DNA content determined from DAPI staining versus DNA content determined from biochemical assay (see Materials and Methods section). The two methods of determining cellular DNA content show good agreement.

C Microscopy images of DAPI-stained DNA and cells. Cellular DNA is localized at single nucleoid foci per cell. This suggests that even in large cells with high cellular DNA content resulting from LacZ OE, only one completely replicated chromosome per cell is present. Therefore, the high cellular DNA content likely results from multiple incomplete replication forks. Moreover, these images demonstrate that cell division proceeds normally in the large cells resulting from LacZ OE (e.g., right image, upper left corner) and the large cell size is not caused by inhibition of cell division as reported under other conditions, for example, by Zaritsky *et al* (2006).

© 2015 The Authors

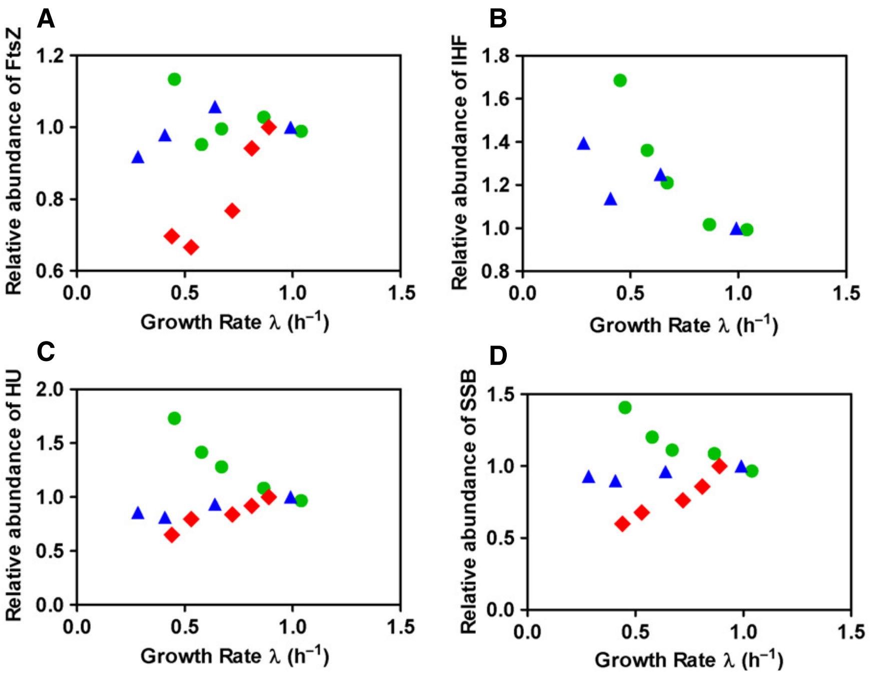

Figure EV4. Relative abundances of exemplary protein levels under the three modes of growth perturbations.
Symbols same as in Fig 1. In the threshold initiator model of cell division (Fig 2A), changes in cell size are reflected by changes in the abundance of the regulatory protein X, ϕ_X. The growth rate dependence of ϕ_X required by our model to produce the observed cell sizes are presented in Fig 2B. Here, we present the relative abundance of some exemplary proteins, as measured in a recent proteomic study (Hui *et al*, 2015), that bear some resemblance to ϕ_X for each mode of growth perturbation. The abundances are normalized to that in the standard glucose condition.

A FtsZ, an essential protein that forms the Z-ring at the site of cell division.

B The DNA-associated protein, IHF, has a matching profile (IHF was not detected in the LacZ OE series, but we know that most proteins exhibit a linear decrease under this perturbation; see Appendix Fig S4). The use of a chromosome-associated protein for cell division control would be an effective strategy to coordinate cell division with the state of chromosome, as has been discussed in the context of eukaryotic cell division (Skotheim, in submission).

C HU, a DNA-binding protein.

D SSB, an essential DNA-binding protein required for DNA replication.

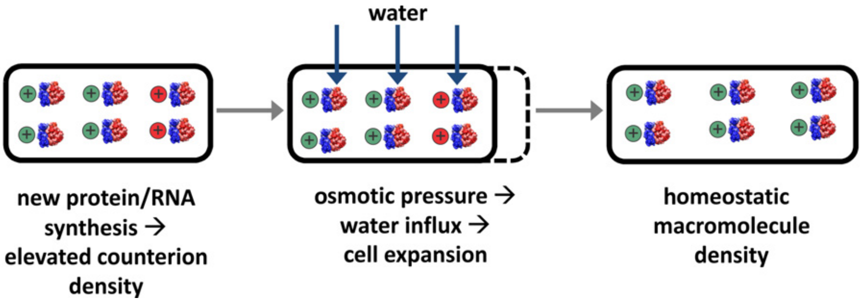

Figure EV5. Chemiosmosis model of biomass–water homeostasis.
Schematic illustration of the proposed chemiosmosis model of biomass–water homeostasis. The observed tight correlation between cellular dry mass (or protein) and cell size (Figs 1E and EV1A) under the different limitations holds even under growth perturbations like LacZ OE, which severely affects the canonical dependence of cell size on growth rate. Since cell size is defined by the sum of cell dry mass and water content, this means the cell keeps nearly a constant ratio of the cellular dry mass and water content. This remarkable coordination of water and dry mass content could be mediated by a homeostatic feedback loop illustrated schematically in this panel: A higher density of charged macromolecules (RNA and proteins) leads to the accumulation of counter ions (e.g., potassium ions and glutamate) via the Gibbs–Donnan effect (Chang, 2005) and thereby results in an increase in intracellular osmolarity (indicated by the red ⊕, left panel). This increase in osmolarity drives water into the cytoplasm, which leads to cell size expansion (dashed line, middle panel), lowering the density of macromolecules back toward the homeostatic level (right panel). Such a mechanism could account for the coordination of cytoplasmic volume with macromolecular content, even under unexpected perturbations like LacZ OE, and is consistent with the recently reported effects of rapid changes of external osmolarity on cell growth (Rojas *et al*, 2014).

 © 2015 The Authors
